# Identification and Assessment of Potential Water Quality Impact Factors for Drinking-Water Reservoirs

**DOI:** 10.3390/ijerph110606069

**Published:** 2014-06-10

**Authors:** Qing Gu, Jinsong Deng, Ke Wang, Yi Lin, Jun Li, Muye Gan, Ligang Ma, Yang Hong

**Affiliations:** 1 Institute of Applied Remote Sensing & Information Technology, Zhejiang University, Hangzhou 310058, Zhejiang, China; E-Mails: funny@zju.edu.cn (Q.G.); linyi11@zju.edu.cn (Y.L.); lijun_more@126.com (J.L.); ganmuye@zju.edu.cn (M.G.); 11114054@zju.edu.cn (L.M.); 2 School of Civil Engineering and Environmental Sciences and School of Meteorology, University of Oklahoma, Norman, OK 73019, USA; E-Mail: yanghong@ou.edu; 3 State Key Laboratory of Hydroscience and Engineering, Department of Hydraulic Engineering, Tsinghua University, Beijing 100084, China

**Keywords:** drinking water, reservoir, water quality, potential impact, CART

## Abstract

Various reservoirs have been serving as the most important drinking water sources in Zhejiang Province, China, due to the uneven distribution of precipitation and severe river pollution. Unfortunately, rapid urbanization and industrialization have been continuously challenging the water quality of the drinking-water reservoirs. The identification and assessment of potential impacts is indispensable in water resource management and protection. This study investigates the drinking water reservoirs in Zhejiang Province to better understand the potential impact on water quality. Altogether seventy-three typical drinking reservoirs in Zhejiang Province encompassing various water storage levels were selected and evaluated. Using fifty-two reservoirs as training samples, the classification and regression tree (CART) method and sixteen comprehensive variables, including six sub-sets (land use, population, socio-economy, geographical features, inherent characteristics, and climate), were adopted to establish a decision-making model for identifying and assessing their potential impacts on drinking-water quality. The water quality class of the remaining twenty-one reservoirs was then predicted and tested based on the decision-making model, resulting in a water quality class attribution accuracy of 81.0%. Based on the decision rules and quantitative importance of the independent variables, industrial emissions was identified as the most important factor influencing the water quality of reservoirs; land use and human habitation also had a substantial impact on water quality. The results of this study provide insights into the factors impacting the water quality of reservoirs as well as basic information for protecting reservoir water resources.

## 1. Introduction

Due to the uneven spatiotemporal distribution of precipitation and the severe deterioration of river water quality, various reservoirs have been functioning for years as the most important sources of drinking water in Zhejiang Province. Reservoirs employed as drinking water sources represent 51% of the total number of centralized drinking water sources in rural areas and 69% in urban areas [1]. The drinking water supply derived from approximately 500 reservoirs supports approximately 70% of the population in Zhejiang Province [2]. Therefore, maintaining the water quality of these reservoirs is particularly important for both water security and socio-economic development at the local and national levels [3]. Monitoring data has indicated that the overall status of the water quality of drinking water source reservoirs is favorable in Zhejiang; however, a number of reservoirs have been subject to increasing pressure and degradation, with a recent deterioration trend [4]. Sewage discharges have increased with the continuing economic development and urban construction, severely damaging the reservoir environment and affecting reservoir function. Simultaneously, as living standards have improved, the demand for high-quality water has also increased [1]. Therefore, a better understanding of the status of the water quality of drinking water reservoirs and the factors that impact water quality is urgently needed.

Identifying the causes of water quality variability is challenging due to the limited availability of data and the absence of a unified theoretical and methodological system, particularly for large-scale studies. Most previous studies have referred to a single or small number of reservoirs [5,6,7] and thus lack generalizability and potential replication. Utilizing sufficient data and effective technologies, we investigated 73 drinking water source reservoirs and attempted to construct a methodological system for analyzing the causes of water quality variability in reservoirs.

Numerous analytical methods have been developed and employed in previous studies to evaluate the factors impacting water quality, such as multivariate analysis [8], artificial neural networks (ANNs) [9], support vector machines (SVMs) [10], and genetic algorithms (GAs) [11]. The relationships between reservoir water quality and impacting factors are generally non-parametric and involve complex interactions. Therefore, favorable model fits are difficult to obtain using traditional statistical methods [12]. Methods using ANNs, SVMs, and GAs may not provide easily understandable explanations for researchers to obtain a complete understanding of the underlying nature of the data [13]. In comparison, decision tree analysis has no distinctive data requirements [14]. It can identify the most decisive variables and offer easily understandable statements. Decision tree analysis has been widely used in various fields, such as ecological modeling, decision making, diagnosis, and marketing operations [15,16,17]. However, it has seldom been applied to water quality studies. In this study, decision tree analysis was employed to classify the water quality levels of reservoirs. The model consists of a set of rules to classify the water quality levels of reservoirs based on independent parameters derived from natural status and anthropogenic activities. In addition, GIS technologies have developed rapidly in recent years and have been successfully applied in various research fields. By contributing to multi-information storage and comprehensive multi-level analysis, GIS technologies have become powerful tools for ecological environmental investigations, particularly those involving wide spatial scales [18,19]. 

Considering the history and current status of water quality and protection measures for drinking-water reservoirs in Zhejiang Province, this paper focuses on the following four main objectives: (1) to establish a decision-making model integrating GIS and classification and regression tree (CART) decision tree analysis for evaluating and analyzing the potential factors impacting reservoir water quality; (2) to identify the parameters that are most closely related to water quality and assess the quantitative importance of these factors on water quality; (3) to analyze the relationships between the identified important parameters and water quality and provide basic information for improving the protection of reservoir water resources.

## 2. Materials and Methods

### 2.1. Study Area

**Figure 1:**
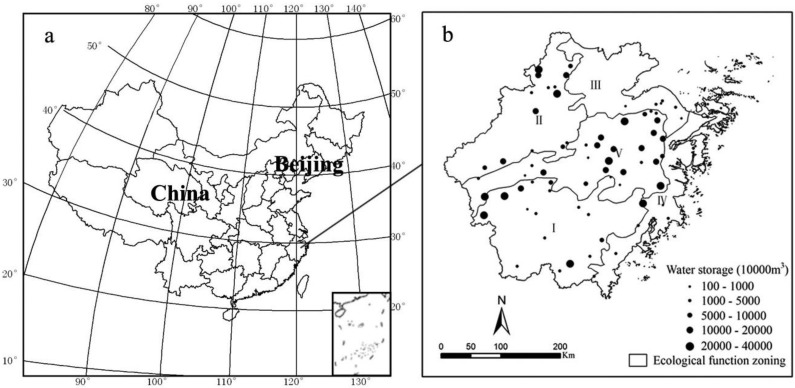
Locations and distribution of drinking-water reservoirs in Zhejiang Province.

Zhejiang Province, which is located in China’s eastern coastal area, is one of the most developed provinces in China. With an area of 101,800 km^2^ and a population of 54.77 million, Zhejiang is also one of the smallest and most densely populated provinces. The terrain in Zhejiang is complex and dominated by mountains and hills, which represent 70.4% of the area of the province. Plains and basins cover 23.2% of the province, whereas rivers and lakes cover 6.4%. Characterized by a subtropical monsoon climate, the region is warm and humid with substantial rainfall, distinct seasons, and sufficient sunlight. The annual average temperature ranges from 15 °C to 18 °C, and the annual average precipitation is between 980 and 2,000 mm. There were 479 reservoirs serving as sources of drinking water in Zhejiang Province in 2010. For this study, we selected 73 reservoirs representing the most important drinking water source reservoirs as an indicator of the overall reservoir status in Zhejiang Province. The locations and relative sizes of the sampled reservoirs are shown in Figure 1. 

### 2.2. Data

**Figure 2:**
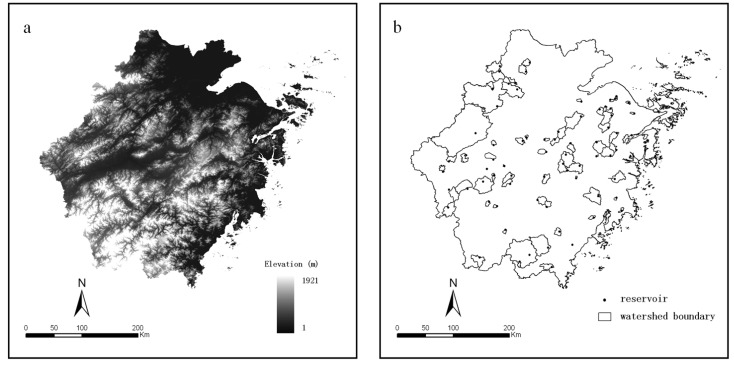
(**a**) DEM of Zhejiang Province; (**b**) boundaries of the watersheds of sampled reservoirs.

The following comprehensive data were used in the sequential evaluation: (1) a digital land use map for the year 2010 provided by the Chinese Ministry of Environmental Protection. The dataset was retrieved from the interpretation of remote-sensing data and field surveys with a validated overall accuracy exceeding 90%; (2) a digital elevation model (DEM) with 30 m resolution (Figure 2a), administrative division, and drainage maps, supplied by the Environmental Science Research Institute of Zhejiang Province; (3) historical water quality monitoring records, reservoir storage capacity and age information supplied by the Zhejiang Environmental Protection Bureau; (4) annual socio-economic data extracted from the Zhejiang Statistical Yearbook (2010) [20]; (5) meteorological records, obtained from the China Meteorological Data Sharing Service System; (6) map of ecological function zoning in Zhejiang Province. According to the ecological function zoning, the provincial terrestrial area is divided into five ecological function zones: southwest mountainous zone (I), northwest mountainous hilly zone (II), northeast plain zone (III), eastern coastal zone (IV) and central hilly basin zone (V) (Figure 1b).

Source data were further processed by the following steps: (1) watershed boundaries were delineated using the DEM in the Hydrology module of ArcGIS 9.3, assisted by the drainage map (Figure 2b); in this process, the parameter of flow accumulation for segmentation was 4,000, the minimum number of cells for a stream was 1,000, and the minimum number of cells for a basin was 2,000; (2) meteorological data were interpreted using the Kriging method; (3) the geo-reference of all layers was unified with the Universal Transverse Mercator (UTM) grid system, WGS_1984 geodetic datum; and (4) the watershed boundary map was overlaid on all other layers to compute parameters within the watersheds using ArcGIS.

### 2.3. CART Decision Tree Model

Decision trees are an important type of data mining algorithm. The basic process of a decision tree is to split a complex decision into a series of simpler decisions [21], potentially producing a solution that is easier to explain. With hierarchical, sequential classification structures, decision tree analysis can extract implicit information from bodies of data by recursively partitioning the learning sets [22,23]. Considering the ability to model the non-linear and non-additive relationships between a dependent variable and a certain number of independent variables, the CART model introduced by Breiman *et al.* [23] was employed to differentiate the water quality levels in this study. The importance of the independent variables relative to the dependent variable can be measured, and closely related independent variables are selected by CART. Moreover, a clearly understandable hierarchical system of decision rules for object classification can be easily displayed [14].

The CART process is sequentially divided into four main steps. The first step is tree building by using recursive splitting of nodes. Beginning with the root node, which includes all samples in the training set, the CART selects the best independent variables to split the node into two descendant nodes. During this process, all possible variables will be tested to find the best splitting values through calculating the maximal homogeneity between the two child nodes. The commonly used “Gini” index is adopted to measure the homogeneity of the two child nodes. Then each node is assigned a predicted class. The node splitting and the assignment of node classes process is repeated for each node whether it is split into descendant nodes and continued recursively. The second step is to stop the tree building process. The tree continuously grows by successive subdivision, which terminates when: (1) only one observation exists in each descendant node; (2) all observations within each of the descendant nodes have exactly the same distribution of independent variables; or (3) the setting of maximal depth in the tree is made by the user in advance. After this step a “maximal” tree has been created, which generally overfits the information contained within the training data. The third step is tree pruning, during which a sequence of simpler trees is generated, by using the method of “cost-complexity” pruning. In this method, a complexity parameter is used to control the pruning process. The fourth step refers to optimal tree selection. The tree which fits the information in the training set, but does not overfit the information, is selected from the series of pruned trees created during the third step. The target in this step, defined in terms of expected performance on an independent dataset, is to find the best complexity parameter so that the information in the training dataset is fit but not overfit. Detailed descriptions of the CART process can be found in [23,24,25,26].

In our decision tree modeling, Forest%, Farmland%, Construction%, DOF, Res_D, Imm_D, GDP, Ind_output, Ind_wastewater, Ind_consumption, Treatment%, Distance, Capacity, and Precipitation were the predictor variables, and the reservoir water quality class in 2010 was the target variable. The CART process was conducted using the software SPSS Clementine 11.1.

### 2.4. Reservoir Water Quality Classes

Referring to the national assessment standards for surface water quality in China (GB3838-2002), all the reservoir water quality was assessed using a single-factor evaluation method and ranged into five classes (named as C1–C5) which were determined by the worst rate of a single index. The employed water quality indices and respective boundary values are described in Appendix A. Based on the monthly provincial monitoring records, each index in a reservoir was averaged by the sampled data. The water quality classes used for the CART process in this study were established based on the monthly mean values of all indices in the year 2010. All the 73 reservoirs meet the drinking water quality demand according to national standard, among which 14, 47 and 12 reservoirs belong to C1 (best class), C2 and C3 respectively.

### 2.5. Comprehensive Impact Assessment Variables for Water Quality Level

By comprehensively considering the regional characteristics of Zhejiang Province as well as the availability, comparability, and reliability of the data, 14 parameters representing anthropogenic activities, reservoir attributes, and climate were selected as independent variables (Table 1). 

**Table 1:** Description ofassessment variables used in the CART analysis.

**Categories**	**Name**	**Abbreviation**	**Unit**
Land use	Percentage of forest	Forest%	
	Percentage of farmland	Farmland%	
	Percentage of construction land	Construction%	
	Degree of fragmentation	DOF	
Population	Resident population density	Res_D	people/km^2^
	Exotic population density	Imm_D	people/km^2^
Socio-economic parameters	Gross domestic product per unit area	GDP	0.1 billion yuan/km^2^·a
	Industrial output value per unit area	Ind_output	0.1 billion yuan/km^2^·a
	Industrial wastewater discharge per unit area	Ind_wastewater	10,000 ton/km^2^·a
	Industrial water consumption per unit area	Ind_consumption	10,000 ton/km^2^·a
	Sewage treatment rate	Treatment%	
Geographical features	Distance to city	Distance	km
	Elevation		m
Characteristics of reservoirs	Storage capacity	Capacity	10,000 m^3^
	Age		year
Climate	Precipitation		mm

Many studies have reported that human habitation and economic activities have a considerable influence on the water quality in adjacent aquatic systems [27,28] because these anthropogenic and economic activities cause emissions of domestic sewage and industrial waste, which threaten the health of the water environment. Based on the procurability of data, we selected Res_D, Imm_D, GDP, Ind_output, Ind_wastewater, Ind_consumption, and Treatment% as independent variables in our study. The resident population density was calculated based on the permanent resident population in the Sixth China Population Census. In addition, considering the status of Zhejiang’s economy, we added immigrant population density as an influencing factor that not only represents human activities but also indirectly reflects the local economic status. The population, GDP, industrial output value, industrial wastewater discharge, and industrial water consumption data were all from the year 2010 and were collected at the administrative scale instead of the watershed scale. Considering the close connection between these parameters and construction land use, we calculated the corresponding amount of each of the above variables for each watershed according to the proportion of construction land in the watershed to that in the administrative region and then used the area of the watershed to calculate the population density and per unit area of the other four variables.

Various investigations have demonstrated that land use has significant impacts on the adjacent hydrologic systems [29,30,31]. The land use patterns are closely linked to the characteristics of anthropogenic activities, which in turn influence the processes by which pollutants are carried into aquatic systems. Water quality in various aquatic systems has been found to be closely related to the compositions of land-use types or spatial configurations of land use patterns within a watershed [31]. In this study, four parameters were employed to represent land use: percentage of forest, percentage of farmland, percentage of construction land, and degree of fragmentation. The degree of fragmentation can reflect the status of the integrity of a terrestrial ecosystem and the conditions of the landscape pattern and is computed as follows:

*C_i_* = *N_i_*/*A_i_* (1)

where Ci is the degree of fragmentation, Ni is the sum of patches, and Ai is the total regional area.

Geographical position reflects the transport processes for pollutants across the landscape and is closely related to local land-use patterns and economic development [29]. Therefore, geographical position has a significant impact on the water quality of reservoirs. We chose elevation to indicate the geographical position of reservoirs. The effect of cities on water quality has been widely discussed [32,33]. In this study, the distance from the reservoir to the city was extracted to assess the influence of cities on reservoir water quality.

Reservoirs with different storage capacities have dissimilar purification abilities, and reservoirs with different ages are associated with unequal risks such as risk of contamination, risk of sediment filling and risk of surrounding development. Therefore, both storage capacity and age could lead to differences in water quality. To explore the relationship between the two variables and reservoir water quality, we employed them for modeling.

Hydrology is demonstrated by many studies to be significantly related to the surface water quality [34,35]. In this study, precipitation was utilized as the indication of hydrological status. Precipitation data for each watershed were obtained through the Kriging interpolation method [36,37] based on monthly records from 18 monitoring points in Zhejiang Province in 2010.

## 3. Results

### 3.1. Spatial Distribution of Water Quality

**Figure 3:**
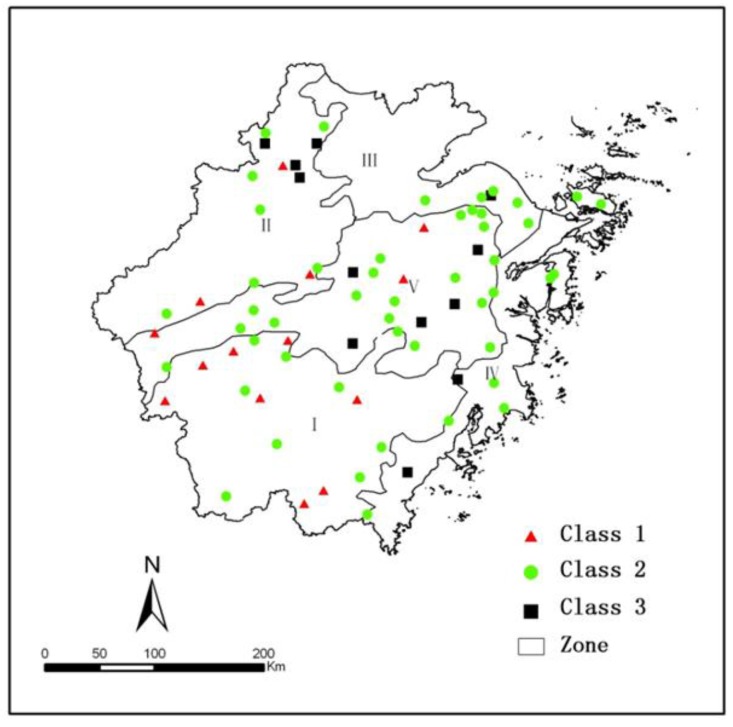
Spatial distribution of reservoir water quality.

The spatial distribution of reservoirs was clearly shown in Figure 3 that most reservoirs with the best water quality (class C1) were intensively located in the southwest mountainous function zone which was dominated with forest and characterized by low social-economic development-intensity, and occurred dispersedly in the northwest mountain-hill zone and central hill-basin zone. The reservoirs with relatively poor water quality (class C3) were dispersedly distributed in all zones except the southwest mountainous zone.

### 3.2. Rules for Predicting Reservoir Water Quality by CART

The 52 training reservoirs were randomly selected from the range of water quality classes and included 10, 34, and eight reservoirs from classes C1, C2, and C3, respectively. The rules (Table 2) included nine nodes and the following seven parameters: Ind_output, Ind_wastewater, GDP, Res_D, Imm_D, Construction%, and Forest%. These parameters primarily represented the surrounding anthropogenic activities, including population agglomeration, land use, and economic activities, and were most closely related to the water quality of the study reservoirs. The remaining parameters were excluded from the rules, not because they had no relation with the water quality of the reservoirs but because they did not significantly affect the water quality compared with the parameters listed above, which masked their effect. This result indicates that the causes of reservoir water quality variability are extremely complex and comprehensive. The accuracy of the CART training process in correctly attributing reservoirs to their respective water quality classes was 94.2%. The testing group was then used to assess the predictive ability of the CART model. The overall CART accuracy for assigning reservoirs to the correct water quality classes was 81.0%, suggesting that the rules for predicting reservoir water quality were acceptable.

**Table 2:** Rules for predicting reservoir water quality classes.

Water Quality Classes	Rules
C1	Ind_output ≤ 0.183 & GDP ≤ 0.195 & Ind_wastewater ≤ 0.119 Ind_output ≤ 0.183 & GDP ≤ 0.195 & Ind_wastewater > 0.119 & Imm_D ≤ 15
C2	Ind_output ≤ 0.183 & GDP ≤ 0.195 & Ind_wastewater > 0.119 & Imm_D > 15 Ind_output ≤ 0.183 & GDP > 0.195 Ind_output > 0.183 & Ind_wastewater ≥ 0.831 & Forest% ≥ 78.1% Ind_output > 0.183 & Ind_wastewater < 0.831 & Construction% ≥ 2.13% & Res_D ≤ 795 Ind_output > 0.183 & Ind_wastewater < 0.831 & Construction% < 2.13%
C3	Ind_output > 0.183 & Ind_wastewater < 0.831 & Construction% ≥ 2.13% & Res_D > 795 Ind_output > 0.183 & Ind_wastewater ≥ 0.831 & Forest% ≤ 78.1%

According to the rules, the reservoir water quality was mainly related to anthropogenic activities. Industrial conditions represented by Ind_output, Ind_wastewater, and Imm_D had a large contribution in separating water quality classes. Ind_output and Ind_wastewater were the most important parameters distinguishing the classes of water quality. The water quality levels of reservoirs located in regions with higher industrial output values were inferior to those of reservoirs in relatively less developed areas. Whereas industrial wastewater discharge clearly poses a great threat to adjacent waters, GDP and population density were also associated with water quality; higher values of both of these parameters increase pressure on reservoirs. In addition, forest and construction land use exerted a certain impact on water quality levels, consistent with the results of [31]. Notably, Farmland% was excluded from the CART rules.

### 3.3. Evaluation of the Influence of Parameters on Reservoir Water Quality

CART could calculate the relative importance of parameters included in the decision tree on water quality classes. However, the variables that did not appear in the rules cannot be quantitatively assessed. To evaluate the influence of parameters on water quality, the 52 training reservoirs were used to obtain the quantitative importance of the parameters Ind_output, Ind_wastewater, GDP, Res_D, Imm_D, Construction%, and Forest%. Table 3 presents the misclassification error of the decision tree model when using different groups of parameters. Parameters related to economic activities including Ind_output, Ind_wastewater, and GDP were most important for differentiating water quality (omission of these parameters increased the misclassification error to 19.2%), followed by land use (misclassification error of 15.4%) and population density (misclassification error of 13.5%). With respect to the influence of a single parameter on reservoir water quality, Ind_wastewater and Ind_output had the greatest influence on water quality, indicating that pollution during industrial production processes is the greatest source of water deterioration. Ind_wastewater and Ind_output were followed by GDP, Construction%, Res_D, Imm_D, and Forest%.

**Table 3:** Misclassification error using different independent variables in CART.

Variables	Misclassification error rate
All	5.8%
Missing Ind_wastewater	17.3%
Missing Ind_output	15.4%
Missing GDP	13.5%
Missing Construction%	13.5%
Missing Res_D	11.5%
Missing Imm_D	9.6%
Missing Forest%	7.7%
Missing Construction%, Forest%	15.4%
Missing Res_D, Imm_D	13.5%
Missing Ind_wastewater, Ind_output, GDP	19.2%

## 4. Discussion

### 4.1. Economic Development and Industrial Pollution in Zhejiang Province

Zhejiang Province is one of the most developed provinces in China and has undergone rapid urbanization and industrialization in recent decades. During this rapid economic growth, neglect of ecological protection and an absence of scientific development concepts have led to excessive consumption of resources and various environmental problems that consequently limit further development and daily life.

The statistical data indicate that from 2006 to 2010, wastewater emissions followed an increasing trend, and the average annual growth rate of industrial wastewater and domestic sewage emissions was 2.5% and 8%, respectively. The wastewater discharge was 3.93 billion tons in 2010, 55.4% of which was from industrial process and the remainder from human activities. Emissions in northern and eastern cities, such as Hangzhou, Ningbo, and Wenzhou, were considerably higher than those in southwestern cities, such as Quzhou, Lishui, and Taizhou, because of the imbalance of development. The discharge of industrial waste gas and solid waste amounted to 2.04 trillion m^3^ and 42.68 million tons in 2010, respectively, representing respective growth rates of 58.9% and 70.5% from the year 2005. Based on the statistics in 2010, for every $100 million of GDP, 0.88 million tons of waste water were discharged, and for every $100 million of industrial output value, 0.88 billion m^3^ of industrial waste gas and 18.4 thousand tons of industrial solid waste were produced, several or even dozens of times higher than the waste produced in developed countries.

### 4.2. Population Density and Water Quality

Human activities produce residential pollutants, primarily food waste, washing residues, hospital sewage, and household garbage [28]. Zhejiang Province is densely populated, and the high population density in reservoir catchments has always been a primary challenge in reservoir water protection. Domestic pollutants with abundant nitrogen and phosphorus and nutrients have increased considerably in recent years with the rapid improvement of people’s living standards. However, due to the lack of proper processing of these pollutants, most were directly discharged into the natural environment and carried by runoff into water bodies [1]. Moreover, the reservoir areas are rich in tourist resources. The dramatic expansion of the catering industry and tourism has led to an increase in the fluid population and thus in pollution.

Thus, it is necessary and urgent to enhance the control of domestic pollutants for reservoir water source maintenance. Construction of sewage treatment facilities is recommended to negate the harmful effects associated with expanding population. Harmless disposal of residential garbage could be an effective way for reservoir water protection. Landfills within watersheds must be strictly prohibited to prevent adverse impact of pollutants on reservoirs. In addition, reductions in population densities within watersheds could be a promising approach to alleviate the pressure on water quality derived from human activities. 

### 4.3. Effects of Land Use on Reservoir Water Quality

Many studies indicate that there is a strong relationship between land use and the water quality in adjacent water bodies [30,31,38]. Consistent with previous studies [30,31], this study demonstrated that the expansion of construction land increases the risk of water quality deterioration, whereas the expansion of forest land benefits adjacent water bodies. The expansion of construction land reflects increased industrial development or population, which lead to increased industrial or domestic pollution.

Agricultural non-point source pollution plays an important role in water quality degradation, which easily occurs on sloping arable land [28]. Unexpectedly, the parameter of percentage of farmland was not selected in the CART model. Baker reported that the extent of negative impact that arable land has on water quality is determined by tillage methods and geographical position [39]. In Zhejiang Province, paddy fields constitute the main arable land, and the farming practices for this type of agricultural land use are different from those for other agricultural land uses in many ways, including fertilization, irrigation, and method of drainage. Jeon and Yoon *et al.* argued that the loading of nutrient from paddies to water bodies is largely determined by the field management of water and fertilizer as well as precipitation [40,41]. To enhance nutrient uptake by plants, farmers keep the paddies flooded after fertilization. Therefore, the negative influence is modest under normal circumstances but is intensified by large amounts of precipitation [40]. In addition, slope plays a significant role in non-point source pollution for paddies or dryland. The arable land in mountain or hilly area has a greater slope, increasing the risk of soil erosion and area-source pollution. However, there is less farmland in these regions compared to plain areas; in the latter, the farmland has a smaller slope, with less risk of pollution. Therefore, the parameter of percentage of farmland does not accurately reflect agricultural non-point source pollution within the watershed.

In conclusion, land use *per se* does not lead to pollution; rather, human activities on the corresponding land determine the types and level of pollution [42]. Measuring the land use in watersheds is an indirect but effective way of projecting human activities and can be used to assess the water quality of the receiving water bodies as long as the characteristics of anthropogenic activity and natural factors in a specific region are considered [31].

### 4.4. Precipitation and Reservoir Water Quality

It is interesting that precipitation was not identified as an important variable in the CART model. It could be attributed to the fact that the precipitation has complicated effect on receiving water quality, with both positive and negative sides. Changes in rainfall could affect surface runoff and reservoir storage and, hence, the mobility and dilution of contaminants [34]. More receiving water can increase the water mobility, benefitting the contaminants dilution, but could possibly bring in more pollutants such as nutrient and heavy metal [29,43]. Additionally, although abundant rainfall benefits the vegetation growth combined with water conservation, it can also increase the risks of soil erosion combined with deterioration in water quality [44]. Therefore, it is difficult to quantitatively evaluate the relation between precipitation and reservoir water quality.

### 4.5. Reservoir Water Quality Protection Based on Ecological Function Zoning

The five primary-level ecological function zones which were partitioned principally based on natural climate, geographical characteristics and landforms, and social-economic development situation have imposed significant impact on drinking-water reservoir protection. However, although specific protection policies such as the optimization and upgrading of industry have been promoted for the northeastern plain zone, central hilly basin zone, and eastern coastal zone, there has been a tendency of industry to shift from the southeast coastal areas to the sparsely populated southwestern areas which has inevitably imposed increasing pressure upon the reservoirs in these areas. Therefore, drinking water source protection requires proper execution of an ecological function zoning strategy and strict regulation of the development and transfer of industry.

In addition, several other types of spatial zoning and regulation focusing on land resource exploitation and configuration, among which urban and rural planning, overall planning for land utilization are of greatest importance to the government will also give rise to potential and specific impact on drinking-water reservoir. In fact, the ecological function zones have acted as the imperative guideline and spatial control boundary for various zoning and regulation in practice.

## 5. Conclusions

In this study, the CART decision tree method was employed to estimate the classes of reservoir water quality based on a set of parameters, and a reasonable accuracy was obtained. The CART analysis indicated that most of the parameters comprising the rules encoded by anthropogenic factors, including industrial activities, human habitation, and land use, are likely responsible for reservoir water quality variability. The quantitative comparison of the importance of the seven parameters included by the rules revealed that industrial emissions were the most important factor for the variability of reservoir water quality. The methodology proposed in this study enables the rapid, robust, and informative identification of the causes of variation of reservoir water quality and is applicable to other areas, potentially serving as an operational tool for planners and managers.

## Appendix A

**Table A1:** Environmental guideline of national quality standards for surface waters, China (GB3838–2002) (units: mg/L).

Parameters		Category of water quality standards				
		First	Second	Third	Fourth	Fifth
DO	≥	7.5	6	5	3	2
COD_Mn_	≤	2	4	6	10	15
COD	≤	15	15	20	30	40
BOD	≤	3	3	4	6	10
NH3-N	≤	0.15	0.5	1	1.5	2
TP	≤	0.01	0.025	0.05	0.1	0.2
TN	≤	0.2	0.5	1	1.5	2
TCu	≤	0.01	1	1	1	1
TZn	≤	0.05	1	1	2	2
F^−^	≤	1	1	1	1.5	1.5
TSe	≤	0.01	0.01	0.01	0.02	0.02
TAs	≤	0.05	0.05	0.05	0.1	0.1
THg	≤	0.00005	0.00005	0.0001	0.001	0.001
TCd	≤	0.001	0.005	0.005	0.005	0.01
Cr^6+^	≤	0.01	0.05	0.05	0.05	0.1
TPb	≤	0.01	0.01	0.05	0.05	0.1
TCN	≤	0.005	0.05	0.2	0.2	0.2
V-ArOH	≤	0.002	0.002	0.005	0.01	0.1
Petroleum	≤	0.05	0.05	0.05	0.5	1
Anionic surfactant	≤	0.2	0.2	0.2	0.3	0.3
S^2−^	≤	0.05	0.1	0.05	0.5	1
Fecal coliform (number/L)	≤	200	2,000	10,000	20,000	40,000
